# Mental distress and well-being of university students amid COVID-19 pandemic: findings from an online integrative intervention for psychology trainees

**DOI:** 10.3389/fpsyg.2023.1171225

**Published:** 2023-07-13

**Authors:** Vasiliki Yotsidi, Eirini-Konstantina Nikolatou, Elias Kourkoutas, Georgios A. Kougioumtzis

**Affiliations:** ^1^Department of Psychology, Panteion University of Social and Political Sciences, Athens, Greece; ^2^Department of Primary Education, Research Center for the Humanities, Social and Education Sciences, University of Crete, Rethymno, Greece; ^3^Department of Turkish Studies, National and Kapodistrian University of Athens, Athens, Greece; ^4^Department of Psychology, Neapolis University Pafos, Pafos, Cyprus

**Keywords:** COVID-19, university students, mental distress, well-being, resilience, risk factors, protective factors, online intervention

## Abstract

**Introduction:**

University students have been severely affected by the COVID-19 pandemic, as significant changes supervened their academic and social life. To tackle these challenges, several adjustments in the educational methods may be warranted for cultivating a positive environment at higher education institutions. The aim of this study was to investigate the risk and protective factors of students’ mental health and well-being as well as their potential for flourishing in an undergraduate clinical psychology course that took place online due to the COVID-19 restrictive measures and incorporated positive psychology exercises as a means to empower young people amid the adverse conditions of the lockdown.

**Methods:**

In total, 124 students attended the course and completed mental health (i.e., DASS-9, HADS, ERQ) and well-being (i.e., MHC-SF, SWLS, PANAS, GQ-6, BRS) measures at two time points (pre-and-post-test), during the first restrictions in Greece (March–June 2020).

**Results:**

According to the results, students aged 18–20 years old reported higher levels of stress [*χ*^2^ = 14.72, *p* = 0.002], while students who felt that the quality of their studies had deteriorated [*χ*^2^ = 6.57, *p* = 0.038] reported increased levels of anxiety. High levels of depression were correlated with worse relationships with significant others (*z* = 7.02, *p* = 0.030 and *χ*^2^ = 11.39, *p* = 0.003 for family and friends, respectively), while gratitude and resilience were positively correlated with improved relationships with others, both during and after the lockdown. Factors associated with students’ well-being were satisfaction with life and gratitude.

**Discussion:**

These results suggest that well-being enhancement factors may have added value to current educational practices for promoting students’ mental health and well-being in times of crisis.

## 1. Introduction

The COVID-19 pandemic and the subsequent restrictive measures imposed detrimental effects on peoples’ mental health and well-being across the globe. Common psychological effects of the COVID-19 disease were reported to include increased levels of uncertainty and helplessness, stress, anxiety, post-traumatic stress disorder (PTSD), psychological distress, depression as well as higher prevalence of harmful behaviors, such as self-injury, suicidal thoughts and behaviors, and substance use (Serafini et al., 2020; Thakur and Jain, 2020; Blasco-Belled et al., 2022; Bonati et al., 2022; Hernández-Díaz et al., 2022; Leung et al., 2022; Pappa et al., 2022). These alarming findings prompted further research on the risk and protective factors correlated with mental distress due to the COVID-19 outbreak, so as to inform policy and practice interventions in order to tackle the adverse consequences of the pandemic in the long-run.

Being young was found to be a main risk factor for being psychologically affected by the pandemic in numerous studies (Ho et al., 2020; Huang and Zhao, 2020; Mazza et al., 2020; Wang et al., 2020b). Other risk factors included female gender (Gurvich et al., 2020), living in a rural area (Lee et al., 2021; Silișteanu et al., 2022), low level of education, poor mental and physical health, and lack of children (Gao et al., 2020; Özdin and Bayrak Özdin, 2020; Smith et al., 2020) as well as being in contact with relatives infected, or suspected infection, with COVID-19 (Cao et al., 2020). On the other hand, protective factors were found to be several social and positive psychological resources. With regard to the latter, high levels of resilience, gratitude, hope, satisfaction with life, and meaning in life (Kavčič et al., 2020; López et al., 2020; Trzebiński et al., 2020; Wang et al., 2020a) as well as positive reframing and humor (Gurvich et al., 2020) were correlated with lower levels of stress, anxiety and depression, and better mental health. Additionally, positive family functioning was found to ameliorate the negative impact of the pandemic and the subsequent quarantines, since experiencing family acceptance and support, increased shared feelings, and caring for and toward others (Gurvich et al., 2020; López et al., 2020; Zhang and Ma, 2020; Wang et al., 2020a) were all found to be acting as a bulwark in these difficult times.

Amidst COVID-19 pandemic, university students seemed to be particularly affected, as they have been faced with disruptions of academic life and social connections that caused them serious mental health effects. Furthermore, many college students had to return to the parental home environment due to the restrictive measures, thus refraining from their strivings for independence. The WHO’s report on the impact of COVID-19 pandemic on mental health and wellbeing (World Health Organization, 2022) highlighted the need for developing age-related interventions for young people, especially young adults aged 20–24 years old who were found to be the most severely affected age-group by COVID-19 and the most susceptible population for serious mental health problems. Indeed, previous studies have shown that university students experienced significant psychological distress during the lockdown periods, reporting low levels of resilience (Alyoubi et al., 2021), loneliness (Holm-Hadulla et al., 2021; Werner et al., 2021; Koelen et al., 2022), high levels of stress and anxiety (Charles et al., 2021; Rogowska et al., 2021; Visser and Law-van Wyk, 2021; Elharake et al., 2022; Fang et al., 2022; Xu and Wang, 2023), depression (Kaparounaki et al., 2020; Evans et al., 2021; Volken et al., 2021; Elharake et al., 2022; Fang et al., 2022; Sauer et al., 2022; Xu and Wang, 2023), psychosomatic symptoms (Hadjicharalambous et al., 2021; Silișteanu et al., 2022), alcohol and substance use disorder (Charles et al., 2021; El-Monshed et al., 2021; Prowse et al., 2021), and suicidal thoughts (Wang et al., 2020; Arsandaux et al., 2021).

The prevalence of psychological distress and mental health problems in university students was found to be determined by several academic factors, including e-learning (Chinna et al., 2021; Yang et al., 2021; Appleby et al., 2022), following theoretical studies (Odriozola-González et al., 2020; Patsali et al., 2020), being at a higher year of study (Silișteanu et al., 2022), perceived academic difficulties (Kecojevic et al., 2020; Piya et al., 2022), academic anxiety (Son et al., 2020; Chinna et al., 2021; Lee et al., 2021), uncertainty related to academic future and career prospects as well as interpersonal conflicts and restrictions in socializing (Padrón et al., 2021). Young people from diverse/minority backgrounds (e.g., Non-Hispanic Black, Black, and sexual minority students) were found to be at higher risk of increased depression symptoms (Fruehwirth et al., 2021; Kim et al., 2021; Zinchenko et al., 2021) compared to their classmates. Similarly, female students exhibited higher levels of stress (Ghazawy et al., 2021; Ochnik et al., 2021; Padrón et al., 2021; Prowse et al., 2021; Talapko et al., 2021), anxiety and depression (Kibbey et al., 2021; Lee et al., 2021; Sun et al., 2021), and worse general mental health (Reyes-Molina et al., 2022). Pre-existing chronic diseases or mental health conditions were an additional risk factor for being severely impacted by the COVID-19 pandemic (Li et al., 2020; Ghazawy et al., 2021). Under the pressure of these unprecedented challenges at a population-level, the academic community was alarmed to be at the forefront to protect young people’s well-being and mental health.

To this end, the integration of students’ well-being into the university curricula may play an important role in curbing the effects of adverse conditions due to the COVID-19 pandemic, given that individuals with high well-being have been found to adopt coping behaviors that promote psychological health (Boehm et al., 2012; Kim et al., 2015). Mental well-being includes a tripartite model of emotional, psychological, and social well-being, and it refers to a state of flourishing –distinct from languishing or moderate mental health (Keyes, 2002, 2005, 2006). Students who flourish have greater self-control and higher academic performance (Datu, 2018). They focus more on their personal development and they are academically and socially more committed, compared to non-flourishing students (Gokcen et al., 2012; Huebner et al., 2014; Heffner and Antaramian, 2016). They also experience higher levels of positive emotions and satisfaction with life (Ouweneel et al., 2011; Van Zyl and Rothmann, 2012), while they are more likely to use adaptive strategies that maintain positive affect (Basson and Rothmann, 2018), such as building social support networks and exhibiting environmental mastery, both of which act as a buffer against stressful and traumatic circumstances (Howell, 2009).

A large body of research has demonstrated that adaptive coping was necessary for sustaining university students’ mental health during the COVID-19 outbreak. In particular, the harmful effects of the pandemic were found to be mitigated when high levels of problem-focused coping strategies (El-Monshed et al., 2021), prevention of negative emotions (Xiong et al., 2021), increased acceptance (Chinna et al., 2021), hope (Yu et al., 2021), reframing skills and daily routines (Padrón et al., 2021), emotional intelligence (Barros and Sacau-Fontenla, 2021), resilience and emotion regulation strategies (Ye et al., 2022; Qin et al., 2023), social support (Cao et al., 2020; Otanga et al., 2021), COVID-19 self-efficacy (Sun et al., 2021) as well as exercising and physical activity (Lee et al., 2021; Reyes-Molina et al., 2022) were reported by students. Although these responses contributed to embracing the COVID-19 challenges, they were not common among young people in higher education. On the contrary, the vast majority of university students presented severely reduced well-being during the pandemic, as many studies have showed (Holm-Hadulla et al., 2021; Zhu et al., 2021; Kokkinos et al., 2022; Mosleh et al., 2022). According to Visser and Law-van Wyk (2021), most of the university students were languishing rather than flourishing in the COVID-19 era. To tackle these negative effects of COVID-19 among young people, several adjustments in the educational methods are warranted for cultivating a positive environment at higher education institutions and addressing mental health issues that have been caused or exacerbated by COVID-19.

In response to concerns around the long-lasting pandemic’s impact on mental health, it has been suggested that more global approaches to cultivating well-being should be taken by academic institutions to boost young people’s coping skills against the negative implications of the COVID-19 and future crises (Grubic et al., 2020). Yet, the number of relevant studies regarding the implementation of well-being programs at university contexts amid the pandemic remain scarce, to date. Morgan and Simmons (2021) have developed an 8-week online positive education program based on Seligman’s (2011) PERMA (positive emotion, engagement, relationships, meaning, and accomplishment) framework to promote well-being in undergraduate and postgraduate psychology students. The results from the delivery of the intervention highlighted the need for ongoing wellbeing support requirements following the pandemic and the wider integration of positive education practices in university contexts (Morgan and Simmons, 2021). Following a two-group pretest-posttest control design on college students, Villarino et al. (2022) found no significant differences and relationships in the pre-test and post-test PERMA scores among the experimental group, after a 7-week online well-being program based on cognitive behavioral therapy (CBT) and PERMA model. Given that well-being is a multifaceted construct, the researchers argued that a holistic approach that would embed well-being frameworks and techniques into the curriculum is likely to be more beneficial than stand-alone interventions in higher education institutions. Such an inclusive approach would minimize systemic inequalities and barriers within the academic community and it would prioritize mental health care for university students along with academic performance demands.

In this context, the aim of this study was to investigate the risk and protective factors of students’ mental health and well-being as well as their potential for flourishing in an online undergraduate psychology course that incorporated positive psychology exercises in the core curriculum, as a means to empower young people amid the adverse conditions of the first restrictive measures in Greece (March–June 2020). Since this was the first lockdown in the country, the academic community struggled to adapt to online teaching in a short period of time. Hence, it was essential to provide students with tools and resources that would promote their well-being, along with supporting them academically in an entirely virtual learning environment, which may have exacerbated already existing academic barriers and strains. As such, an inclusive 8-week course was delivered based on PERMA framework and Keyes’ well-being model of mental health (2002, 2005, 2006). Adopting a bottom-up method of the “whole-university” approach (Hoare et al., 2017), the selection of the particular well-being components to be embedded into the curriculum was also based on the results regarding the risk and protective factors of students’ mental health and well-being assessment conducted at the beginning of the semester. At the end of the course, the same factors were reassessed to discern any pre-and-post differences in mental health and well-being among the student population. Thus, the main research questions of the study pertain to the following: (a) which are the risk and protective factors of university students’ mental health (stress, anxiety and depression) and well-being during the first restrictive measures in Greece among psychology undergraduates, and (b) how positive psychology factors may affect students’ mental health and well-being after incorporating well-being enhancement into current educational practices in a psychology course.

## 2. Method

### 2.1. Study design

This uncontrolled nonrandomized trial used a convenience sample of undergraduate psychology students at the Department of Psychology of a state university in Athens, Greece (i.e., Panteion University of Social and Political Sciences) in a pretest-postest design. During the first COVID-19 restrictive measures, we invited all the undergraduate psychology students who were enrolled in an optional clinical psychology course for the second semester of the academic year 2020–2021 to answer a survey and to participate in the 8-week online well-being enhancement course that integrated a multi-component approach (Carr et al., 2021; Koydemir et al., 2021), with mental wellbeing as the primary outcome based on Keyes’ tripartite model (2002, 2005, 2006). The only additional inclusion criterion for participation was to provide written informed consent. Participation in the study was on a voluntary basis. The study also had an exploratory component that investigated correlates for wellbeing and mental health among psychology undergraduates during the first restrictive measures in Greece, with the aim to assess specific risk and protective factors in line with current empirical evidence (e.g., Elharake et al., 2022; Fang et al., 2022; Xu and Wang, 2023).

### 2.2. Participants

The participants were 1st year to 5th year psychology students. From the total number of 139 students who had enrolled in the course, 126 students provided informed consent and completed the pre-test online survey. Two of these students withdrew from the course after the first assessment due to personal reasons. Thus, data from 124 students who attended the optional clinical psychology course and participated in both the pre-test and post-test measurements of the study were available for analysis. The majority of the participants were female, aged between 18 and 30 years old, single without children, and their home of residence was in an urban area (Table 1). Almost half of them (47.6%) lived with their parents. A small number of students (*n* = 24) were graduates from other scientific fields, prior to the current studies in psychology. The vast majority of participants (93.6%) reported excellent to good physical health at the time of the study. Only two students belonged to a high-risk group for COVID-19 severe infection, while 39.5% of the participants lived with persons who were at increased risk for being infected by COVID-19 due to serious pre-existing health problems.

**Table 1:** Sociodemographic profile of the participants.

*Demographic characteristics*		*Ν*	*%*
Sex	Male	10	8.1
	Female	114	91.9
Age	18–20	49	39.5
	21–30	57	46.0
	31–40	11	8.9
	> = 41	7	5.6
Marital status	Married	4	3.2
	Unmarried	109	87.9
	Divorced	4	3.2
	Other	7	5.6
Children	Yes	3	2.4
	No	121	97.6
Place of residence	Urban	108	87.1
	Semi-urban	15	12.1
	Rural	1	0.8
Conditions of residence	Alone	31	25.0
	With friends/roommate	7	5.6
	With parents	59	47.6
	With partner/spouse	9	7.3
	With partner/spouse and kid(s)	1	0.8
	With kid(s) and parents	1	0.8
	Other	16	12.9
Nationality	Greek	120	96.8
	Other	4	3.2
Level of education	High school graduate	100	80.6
	Higher education graduate	24	19.4
Academic year	1st	30	24.2
	2nd	18	14.5
	3rd	27	21.8
	4th	38	30.6
	> = 5th	11	8.9

### 2.3. Materials

#### 2.3.1. Mental health measures

##### 2.3.1.1. Depression anxiety stress scale (DASS-9)

DASS-9 is a short self-report questionnaire developed by Yusoff (2013) to measure mental distress. It contains 9 items to assess depression, anxiety, and stress (i.e., 3-items per dimension) on a 4-point Likert-type scale (0 = did not apply to me at all, 3 = applied to me very much, or most of the time). DASS-9 has shown good reliability and validity in the Greek context (Kyriazos et al., 2018b). In our study, Cronbach’s *α* was 0.77, 0.64, and 0.61 for depression, anxiety, and stress, respectively.

##### 2.3.1.2. Hospital anxiety and depression scale (HADS)

HADS is a 14-item screening tool consisting of two subscales for anxiety and depression symptomatology, respectively (Zigmond and Snaith, 1983). Each item is rated on a 4-point Likert scale ranging from 0 to 3. Total subscale scores of more than 8 points denote considerable severity of symptoms. Cronbach’s alphas range from 0.68 to 0.93 for anxiety and from 0.67 to 0.90 for depression (Bjelland et al., 2002). HADS has been validated in Greece (Michopoulos et al., 2008). In our study, Cronbach’s alpha was 0.81 for anxiety and 0.73 for depression.

#### 2.3.2. Well-being measures

##### 2.3.2.1. Mental health continuum-short form (MHC-SF)

Mental well-being was measured by the MHC-SF (Keyes et al., 2008), which is a widely used questionnaire to evaluate three facets of well-being: emotional, psychological, and social (Keyes, 2002). It contains 14 items rated on a 6-point Likert scale (0–5) with higher scores suggesting higher frequency of well-being experiences during the past month. The internal consistency of MHC-SF total and subscales was found satisfactory (Lamers et al., 2011). The Greek version of the MHC-SF was valid and reliable for monitoring well-being in both clinical and nonclinical samples (Ferentinos et al., 2019). In our study, Cronbach’s alphas were 0.91 for the total MHC-SF, and 0.87, 0.87, and 0.80 for emotional, psychological and social well-being, respectively.

##### 2.3.2.2. Positive and negative affect schedule (PANAS)

The PANAS questionnaire (Watson et al., 1988) measures trait affective states, comprising two 10-item mood subscales of positive and negative affect (PA and NA, respectively). Responses are rated on a Likert scale ranging from one to five (i.e., “very slightly or not at all” to “very much”). Cronbach’s alphas range from 0.86 to 0.90 for PA and from 0.84 to 0.87 for NA. In the present study, using a validated Greek version (Daskalou and Sigkollitou, 2012), Cronbach’s alphas were 0.81 and 0.83 for the positive and the negative affect, respectively.

##### 2.3.2.3. Satisfaction with life scale (SWLS)

SWLS is a well-known 5-item scale to measure life satisfaction (Diener et al., 1985). Respondents use a 7-point Likert scale (i.e., from “strongly disagree” to “strongly agree”) with higher scores indicating greater perceived satisfaction. Cronbach’s alpha ranges from 0.79 to 0.89 (Pavot and Diener, 1993). In our study, using a validated Greek version (Lyrakos et al., 2013), Cronbach’s *α* was 0.87.

##### 2.3.2.4. Brief resilience scale (BRS)

The BRS scale evaluates the ability to recover from adversity (Smith et al., 2008). It contains 6 items rated on a 5-point Likert scale (i.e., from “strongly disagree” to “strongly agree”). High scores denote greater psychological resilience. Cronbach’s alpha ranges from 0.80 to 0.91. In our study, we used the Greek validated version (Kyriazos et al., 2018a), which showed satisfactory reliability (*α* = 0.85).

##### 2.3.2.5. Gratitude questionnaire (GQ-6)

The experience of gratitude was assessed by the 6-item Gratitude Questionnaire (McCullough et al., 2002). Responses are rated on a Likert scale ranging from one to five (i.e., “strongly disagree” to “strongly agree”). Cronbach’s alpha is 0.82. In the present study, Cronbach’s alpha was 0.73.

##### 2.3.2.6. Emotion regulation questionnaire (ERQ)

The ERQ questionnaire is a 10-item scale that measures two common strategies for emotion regulation: cognitive reappraisal and expressive suppression (Gross and John, 2003). Respondents use a 7-point Likert scale (i.e., from “strongly disagree” to “strongly agree”). Cronbach’s alpha is 0.79 for cognitive reappraisal and 0.73 for expressive suppression. In our study, the internal consistency was satisfactory for both subscales (i.e., *α* = 0.90 and *α* = 0.84 for cognitive reappraisal and expressive suppression, respectively).

#### 2.3.3. Socio-demographic, relational, and academic data

Socio-demographic variables were collected for all participants including questions about gender, age, education, marital status, children, place of residence, conditions of living, and year of study. Participants were also asked to self-rate their physical health as excellent, very good, good, fair, or poor. Questions about whether participants themselves, or people with whom they lived together, belonged to a high-risk group for COVID-19 severe infection were also included.

Furthermore, the impact of the first lockdown was assessed through questions with regard to the perceived quality of academic studies and relationships with others after the COVID-19 outbreak compared to the period prior to the pandemic. In particular, participants were asked to rate in a 5-point Likert scale (i.e., “worse,” “rather worse,” “stable,” “rather improved,” “improved”) the following aspects of family, social, and academic life: (a) family relationships, (b) their relationship with spouse/partner, if any, (c) relationships with friends, (d) relationships with classmates, and (e) the quality of academic studies, given that this was the first time that teaching was delivered online.

### 2.4. Procedure

After the announcement of the first lockdown measures in the country on March 10, 2020, an unanticipated transition of all academic courses into online teaching took place, which continued even during the university’s examination and assessment period, long after the end of the nationwide lockdown. In response to these unprecedented academic challenges, an 8-week online well-being enhancement course that incorporated positive psychology exercises in the core curriculum of the traditional undergraduate course, entitled “Therapist-client relationship in clinical settings,” was organized from 14/4/2020 to 10/6/2020. The duration of this integrative course was based on previous evidence regarding the impact of the length of sessions on the effectiveness of positive psychology interventions (Bolier et al., 2013; Carr et al., 2021; Koydemir et al., 2021).

Although the course initially began in person for 2 weeks, it moved to online delivery through MS Teams virtual learning environment up to the end of the semester. This is an optional clinical psychology course of the second semester delivered within the Department of Psychology at the Panteion University of Social and Political Sciences (Athens, Greece). Such courses are organized into 13, weekly, 3-hs sessions. The curriculum of the course focuses on the main theoretical concepts, clinical challenges, and therapeutic skills with regard to the therapist-client relationship in the therapeutic work with different clinical populations. In line with recent evidence about the effectiveness of well-being interventions (Carr et al., 2021; Koydemir et al., 2021), the well-being enhancement program incorporated a multi-component approach into the core curriculum of the course, containing the following concepts: (1) positive emotions, (2) accomplishment, (3) self-acceptance and self-compassion, (4) meaning in life and gratitude, (5) positive relationships, (6) engagement and flow, (7) personal growth, and (8) resilience. The weekly sessions began with a thorough presentation of the clinical theme and a brief description of the positive psychology construct. Theoretical, empirical, and practical insights were also provided to promote a comprehensive perspective of well-being constructs in the clinical domain (Yotsidi, 2020). All sessions were delivered by the principal researcher. Participants were encouraged to contribute synchronously to the session with their thoughts and experiences on the theoretical and practical integration of these concepts into the clinical milieu. At the end of each session, reflective homework with well-being activities was provided to the participants to take away with them with the task to present them in a written portfolio.

Prior to the beginning of the integrated well-being course, the participants were informed that their participation was on a voluntary basis and without compensation. They also had enough time to decide whether to continue this course or to enroll in a different optional one, as they had this opportunity according to the academic program. Two measures were completed in the week preceding, and the week following the completion of the 8-week online course. We distributed questionnaires electronically via Google Forms ^©^. Prior to data collection, participants signed informed consent and they were free to withdraw from the study at any time. The completion of the questionnaires was realized with a personal anonymous code created by each student to ensure confidentiality of data, while enabling comparisons between the two different measurements. Participants were also informed that the data gathered would only be used for research purposes.

### 2.5. Statistical analyses

Descriptive results contained absolute and relative (%) frequencies for the qualitative variables, and means and standard deviation (*SD*) for the quantitative variables. Both parametric and non-parametric tests (*t*-test, ANOVA) were used in the examination of the factors correlated with university students’ mental health and well-being for each period under investigation and between them (paired *t*-tests). Additionally, in order to examine the factors determining university students’ well-being prior and after the well-being enhancement course, multiple linear regression model was used and hypothesis tests were performed. In particular, a dashed diagram, Pearson’s *r* and Spearman rank order correlation coefficients were used to measure linear correlation between dependent and independent variables as well as Shapiro–Wilk *W* test for error normality. Furthermore, White’s test for heteroskedasticity, correlogram (ACF plot) and Durbin-Watson test for autocorrelation of residuals as well as Variance Inflation Factor (VIF) for multicollinearity were all employed. For the statistical data analysis PASW Statistics 18 and STATA SE 11 software were used.

### 2.6. Ethics statement

The study was given ethical approval by the Ethics and Deontology Committee of Panteion University of Social and Political Sciences (Protocol number: EHDE/87-240223/YOT). The participants provided their written informed consent to participate in this study. Participation was voluntary and participants did not receive any compensation.

## 3. Results

### 3.1. Preliminary analyses

Table 2 shows the means and SDs of the key study variables at the pre-test and post-test measurements. Although the mean scores of stress and anxiety were higher at post-test, depression was decreased (*M* = 4.92, *SD* = 3.36). At the same time, increases in mean scores were reported in cognitive reappraisal (*M* = 30.74, *SD* = 6.36), positive affect (*M* = 34.14, *SD* = 6.4), psychological well-being (*M* = 20.94, *SD* = 5.28), and total well-being (*M* = 43.6, *SD* = 11.61) at the post-test.

**Table 2:** Means, range and standard deviations of the main study variables pre-test and post-test.

Study variables		Pre-test				Post-test			
		*M*	*Min.*	*Max.*	*SD*	*M*	*Min.*	*Max.*	*SD*
Satisfaction with life	23.42	5	35	5.764	24.11	5	34	5.594
Gratitude	33.23	17	42	5.157	33.36	15	42	5.417
Resilience	3.31	1	5	0.780	3.33	1	5	0.803
Well-being	
Total well-being	42.02	18	67	12.040	43.56	15	70	11.605
Emotional well-being	9.59	2	15	2.908	10.07	3	15	2.889
Social well-being	12.48	2	24	5.185	12.56	2	25	5.157
Psychological well-being	19.96	6	30	5.682	20.94	6	30	5.280
Positive and negative affect	
Positive affect	33.07	15	49	6.371	34.14	17	49	6.404
Νegative affect	25.52	11	44	6.777	25.99	11	44	7.171
Depression, anxiety and stress	
Depression (DASS 9)	2.85	0	9	2.201	2.61	0	9	2.343
Anxiety (DASS 9)	1.84	0	7	1.902	2.14	0	9	2.300
Stress (DASS 9)	3.85	0	9	2.032	4.19	0	9	2.023
Hospital anxiety and depression	
Depression (HADS)	6.18	0	17	3.362	4.92	0	16	3.358
Anxiety (HADS)	6.78	0	16	3.617	6.87	0	19	4.237
Emotional regulation	
Cognitive reappraisal	29.70	9	42	6.822	30.74	8	42	6.360
Expressive suppression	12.04	4	25	5.198	12.19	4	28	5.355

*N* = 124.

Spearman’s rank correlation was computed to assess the relationship between students’ mental health and well-being scores at pre-test and post-test. As shown in Table 3, at the baseline measurement, the participants’ well-being was found to be positively correlated with satisfaction with life, resilience, and gratitude, while it was negatively correlated with depression. The negative correlation between depression and well-being also remained at the post-test measurement. Based on the results depicted in Table 4, the factors that were positively correlated with students’ well-being at the post-test were found to be positive affect and satisfaction with life.

**Table 3:** Spearman’s rho correlations of the main study variables pre-test.

Pre-test															
	SWLS	GQ	BRS	MHC	EWB	SWB	PWB	PA	ΝA	CR	ES	DD	DA	DS	HD
GQ	0.424**														
BRS	0.329**	0.500**													
MHC	0.571**	0.576**	0.546**												
EWB	0.485**	0.431**	0.407**	0.826**											
SWB	0.517**	0.490**	0.472**	0.890**	0.637**										
PWB	0.498**	0.603**	0.564**	0.888**	0.664**	0.649**									
PA	0.289**	0.387**	0.522**	0.454**	0.387**	0.384**	0.460**								
ΝA	−0.239**	−0.266**	−0.424**	−0.382**	−0.367**	−0.274**	−0.397**	−0.171							
CR	0.248**	0.340**	0.300**	0.288**	0.322**	0.227*	0.243**	0.202*	−0.230*						
ES	−0.305**	−0.388**	−0.300**	−0.278**	−0.211*	−0.162	−0.345**	−0.258**	0.167	−0.028					
DD	−0.256**	−0.285**	−0.325**	−0.541**	−0.582**	−0.356**	−0.520**	−0.391**	0.462**	−0.252**	0.250**				
DA	−0.159	−0.402**	−0.386**	−0.358**	−0.304**	−0.252**	−0.413**	−0.330**	0.525**	−0.197*	0.206*	0.366**			
DS	−0.153	−0.341**	−0.369**	−0.435**	−0.449**	−0.370**	−0.399**	−0.163	0.553**	−0.056	0.092	0.434**	0.548**		
HD	−0.227*	−0.245**	−0.319**	−0.485**	−0.571**	−0.339**	−0.433**	−0.469**	0.396**	−0.234**	0.146	0.600**	0.365**	0.297**	
HA	−0.138	−0.195*	−0.464**	−0.306**	−0.321**	−0.220*	−0.290**	−0.213*	0.683**	−0.118	0.223*	0.389**	0.621**	0.600**	0.369**

SWLS, Satisfaction with life; GQ, Gratitude; BRS, Resilience; MHC, Well-being; EWB, Emotional Well-being; SWB, Social Well-being; PWB, Psychological Well-being; PA, Positive Affect; ΝA, Negative Affect; CR, Cognitive Reappraisal; ES, Expressive Suppression; DD, Depression (DASS9); DA, Anxiety (DASS9); DS, Stress (DASS9); HD, Depression (HADS); HA, Anxiety (HADS).

*p < 0.05, **p < 0.01.

**Table 4:** Spearman’s rho correlations of the main study variables post-test.

Post-test															
	SWLS	GQ	BRS	MHC	EWB	SWB	PWB	PA	ΝA	CR	ES	DD	DA	DS	HD
GQ	0.477**														
BRS	0.269**	0.351**													
MHC	0.692**	0.493**	0.388**												
EWB	0.675**	0.401**	0.264**	0.835**											
SWB	0.549**	0.427**	0.366**	0.844**	0.582**										
PWB	0.647**	0.472**	0.353**	0.857**	0.716**	0.524**									
PA	0.350**	0.274**	0.425**	0.531**	0.496**	0.342**	0.568**								
ΝA	−0.404**	−0.339**	−0.383**	−0.423**	−0.415**	−0.296**	−0.415**	−0.168							
CR	0.289**	0.344**	0.344**	0.352**	0.283**	0.299**	0.356**	0.261**	−0.432**						
ES	−0.282**	−0.470**	−0.219*	−0.361**	−0.345**	−0.234**	−0.385**	−0.306**	0.121	−0.124					
DD	−0.311**	−0.313**	−0.369**	−0.540**	−0.471**	−0.376**	−0.553**	−0.550**	0.384**	−0.269**	0.340**				
DA	−0.200*	−0.285**	−0.371**	−0.280**	−0.224*	−0.155	−0.349**	−0.274**	0.482**	−0.302**	0.243**	0.412**			
DS	−0.126	−0.236**	−0.311**	−0.241**	−0.190*	−0.195*	−0.199*	−0.272**	0.456**	−0.14	0.217*	0.480**	0.509**		
HD	−0.451**	−0.301**	−0.410**	−0.599**	−0.623**	−0.408**	−0.562**	−0.622**	0.453**	−0.293**	0.309**	0.616**	0.368**	0.335**	
HA	−0.325**	−0.258**	−0.453**	−0.376**	−0.360**	−0.299**	−0.346**	−0.299**	0.674**	−0.382**	0.130	0.366**	0.649**	0.416**	0.509**

SWLS, Satisfaction with life; GQ, Gratitude; BRS, Resilience; MHC, Well-being; EWB, Emotional Well-being; SWB, Social Well-being; PWB, Psychological Well-being; PA, Positive Affect; ΝA, Negative Affect; CR, Cognitive Reappraisal; ES, Expressive Suppression; DD, Depression (DASS9); DA, Anxiety (DASS9); DS, Stress (DASS9); HD, Depression (HADS); HA, Anxiety (HADS).

*p < 0.05, **p < 0.01.

### 3.2. Mental health, well-being, and sociodemographic variables

During the first lockdown in the country, university students aged 18–20 years old reported higher levels of stress (*χ^2^*_(3)_ = 14.72, *p* = 0.002), while those who were living alone reported higher levels of anxiety (*z* = −2.87, *p* = 0.004). On the other hand, students aged over 40 years old stated increased levels of resilience [*χ^2^*_(3)_ = 10.73, *p* = 0.013], cognitive reappraisal (*z* = −2.31, *p* = 0.020), psychological well-being and social well-being (*χ^2^*_(3)_ = 10.07, *p* = 0.018 and *χ^2^*_(3)_ = 13.31, *p* = 0.004, respectively), during the COVID-19 outbreak.

At the post-lockdown period, young students aged 18–20 years old remained with increased levels of stress (*z* = −2.38, *p* = 0.017), while they also reported higher levels of depression (*χ^2^*_(3)_ = 11.15, *p* = 0.011) than older students. Higher levels of depression were also reported by participants living with their parents after the removal of the restrictive measures (*χ*^2^_(5)_ = 13.09, *p* = 0.023), while those who were living alone (*z* = −2.18, *p* = 0.029) reported higher levels of expressive suppression as an emotion regulation mechanism. Moreover, students who were non-married (*z* = −2.3, *p* = 0.022) and with no children (*z* = −2.38, *p* = 0.017) reported increased levels of anxiety at post-lockdown, compared to the other participants in the study.

Similarly, lower levels of resilience were reported by university students who were between 18 to 30 years old (*F*_(3,120)_ = 4.25, *p* = 0.007). On the other hand, being female was correlated with higher levels of resilience (*t* = 2.19, *p* = 0.030) and expressive suppression (*z* = −2.18, *p* = 0.029), while those students who were living in a semi-urban area (*χ*^2^_(2)_ = 6.29, *p* = 0.043) reported higher levels of social well-being during the post-lockdown period.

### 3.3. Mental health, well-being, and academic variables

University students who felt that the quality of their studies had been deteriorated during the lockdown (*χ*^2^_(2)_ = 6.57, *p* = 0.038) presented increased levels of anxiety. Those students who were graduates from other academic fields prior to their current studies reported higher levels of emotional, social, and psychological well-being [(*z* = −2.24, *p* = 0.025), (*z* = −3.58, *p* < 0.001), and (*z* = −3.56, *p* < 0.001), respectively] as well as higher levels of positive affect (*t* = −2.95, *p* = 0.025), gratitude (*z* = −2.04, *p* = 0.041) and resilience (*z* = −3.47, *p* = 0.001), compared to participants who were studying at Higher Education for the first time.

The latter reported increased levels of negative affect (*t* = 2.38, *p* = 0.019), depression (*z* = −4.25, *p* < 0.001), stress (*z* = −3.16, *p* = 0.002), and anxiety (DASS-9: *z* = −2.90, *p* = 0.004, and HADS: *z* = −2.18, *p* = 0.029) at the period after the lockdown. Mental health problems were also found to be correlated with the year of studies. In particular, students who were in due graduation (i.e., after the 4th year of studies) reported increased levels of depression (*χ*^2^_(4)_ = 10.3, *p* = 0.036). Similarly, students who were at the fifth or further year of studies (*M* = 2.65, *SD* = 0.83) stated lower levels of resilience [*F*_(4, 119)_ = 4.54, *p* = 0.002] compared to respondents who were at the second (*M* = 3.52, *SD* = 0.63), third (*M* = 3.64, *SD* = 0.67), or fourth year of their studies (*M* = 3.4, *SD* = 0.67). Again, graduate students prior to their current studies were found to have better mental health, since they reported higher levels of positive affect (*t* = −2.49, *p* = 0.014), gratitude (*z* = −2.22, *p* = 0.026), resilience (*t* = −3.26, *p* = 0.001), psychological well-being (*z* = −2.54, *p* = 0.011) and total well-being (*z* = −2.19, *p* = 0.029).

### 3.4. Mental health, well-being, and relational variables

Higher levels of depression were reported by university students whose relationships with significant others had been deteriorated during and after the lockdown. Particularly, high levels of depression were correlated with worse relationships with family (*z_(2)_* = 7.02, *p* = 0.030), friends (*χ*^2^ = 11.39, *p* = 0.003) and partner (*χ*^2^_(2)_ = 13.58, *p* = 0.001) during COVID-19 related restrictions. Additionally, deterioration of relationships with friends was correlated with lower levels of positive affect (*F*_(2,120)_ = 12.94, *p* < 0.001) and higher levels of stress (*χ*^2^_(2)_ = 8.29, *p* = 0.016). Again, high levels of depression were correlated with deterioration of family relations at the period after the quarantine, based on the results from DASS-9 (*χ*^2^_(2)_ = 6.44, *p* = 0.040) and HADS (*χ*^2^_(2)_ = 6.2, *p* = 0.031) respective subscales.

On the other hand, improved relationships with family while the quarantine measures were into place were found to be correlated with cognitive reappraisal (*z*_(2)_ = 8.33, *p* = 0.016), gratitude (*z*_(2)_ = 11.24, *p* = 0.004), resilience (*z*_(2)_ = 8.76, *p* = 0.013), and well-being (*z_(2)_* = 16.07, *p* < 0.001). Furthermore, students reported increased levels of well-being when their relationships with classmates were experienced to be improved (*χ^2^*_(5)_ = 14.61, *p* = 0.012). Similarly, they reported more resilience (*χ^2^*_(2)_ = 11.78, *p* = 0.003), and emotional (*χ^2^*_(2)_ = 16.20, *p* < 0.001), social (*χ^2^*_(2)_ = 6.34, *p* = 0.042), and psychological (*χ^2^*_(2)_ = 18.09, *p* < 0.001) well-being when the relationships with their friends were also experienced as improved despite the restrictive measures. After the lockdown, an improved relationship with family (*χ^2^_(2)_* = 6.69, *p* = 0.035), friends (*χ^2^_(2)_* = 9.18, *p* = 0.010), and classmates (*χ^2^_(4)_* = 15.96, *p* = 0.003) was associated with higher levels of students’ gratitude.

### 3.5. Pre-and-post-test differences in mental health and well-being variables

The results of the paired *t*-tests and Wilcoxon signed-rank tests regarding mental health and well-being of participants prior and after the online integrative course also indicated statistically significant differences. Particularly, students’ positive affect (*t* = −2.1, *p* = 0.037), emotional well-being (*Z* = −2.167, *p* = 0.030) and psychological well-being (*Z* = −2.59, *p* = 0.010) as well as satisfaction with life (*Z* = −2.02, *p* = 0.044) were all found to be increased at the post-test compared to the pre-test. Conversely, depression (*Z* = −3.99, *p* < 0.001) was found to be decreased after the implementation of the online well-being enhancement course.

### 3.6. Factors associated with university students’ well-being amid COVID-19

A multiple linear regression model was used to assess the various sociodemographic, relational, and mental health factors that may determine university students’ well-being prior and after the online well-being enhancement course amid the COVID-19 pandemic. Particularly, an equation was examined, where “i” was the individual and “t” was the period of time (pre-test and post-test), consisting of (1) a group of sociodemographic variables (PS) including gender, age, level of education, year of studies, and living alone or not, (2) a group of variables related to family, peer and partner relationships (R), and (3) a group of variables related to mental health (MH) including scores of depression, stress, and anxiety, satisfaction with life, gratitude, resilience, positive and negative affect as well as cognitive reappraisal and expressive suppression, as follows:


$$y_{i,t}^{*}=a+\beta PS_{i,t}+\gamma R_{i,t}+\delta HC_{i,t}+\zeta MH_{i,t}+\varepsilon_{i,t}$$

Table 5 shows the statistically significant results from the method of ordinary least squares (OLS) regression regarding students’ well-being both prior and after the online integrative course. According to the results, mental health and positive psychology factors played an important role on university students’ well-being during the COVID-19 outbreak. Particularly, a one standardized unit increase in depression was associated with decreased well-being by a 0.91 unit at the pre-test and a 0.74 unit at the post-test. On the other hand, a one standardized unit increase in participants’ satisfaction with life was associated with increased well-being in both pre-test and post-test measurements (0.67 and 1.08 units, respectively). Furthermore, a one standardized unit increase in the score of gratitude was associated with increased well-being by a 0.51 unit at the pre-test during the initial phase of the restrictive measures.

**Table 5:** Multiple linear regression results on students’ well-being pre-test and post-test.

	Well-being					
Variables	Pre-test			Post-test		
	*Coefficient*	*95% CI*		*Coefficient*	*95% CI*	
		*LL*	*UL*		*LL*	*UL*
Satisfaction with life	0.6696***	0.3719	0.9673	1.0810***	0.7464	1.4157
	(0.150)			(0.168)		
Gratitude	0.5079*	0.1176	0.8981	0.0693	−0.2913	0.4299
	(0.196)			(0.182)		
Depression (HADS)	−0.9146**	−1.5521	−0.2771	−0.7387*	−1.3743	−0.1031
	(0.321)			(0.320)		
*Ν*	123			124		
*Anova*	*F(30, 92)* = 8.26, *p* = 0.000			*F(30, 93)* = 9.94, *p* = 0.000		
*R-squared*	0.7293			0.7622		
*Adj R-squared*	0.6411			0.6855		
White’s test for homoskedasticity	*χ^2^*_(122)_ = 123, *p* = 0.4576			*χ^2^*_(123)_ = 124, *p* = 0.4578		
Shapiro–Wilk *W* test for normal data	*W* = 0.98870, *p* = 0.40719			*W* = 0.99179, *p* = 0.67917		
Mean VIF	2.89			3.02		

Standard errors in parenthesis. ***p < 0.001, **p < 0.01, *p < 0.05.

## 4. Discussion

Mental distress and mental health conditions in higher education students have already been increasing in the last few years prior to the pandemic (Pedrelli et al., 2015; Auerbach et al., 2018). These pre-existing issues in university students’ mental well-being have subsequently been exacerbated by the pandemic (World Health Organization, 2022), since the academic systems faced several challenges in a very short timescale. The closure of universities, the abrupt transition to remote teaching, the social isolation, and the unforeseen return of many students to their parents’ home, they all were additional stressors that impacted higher education students, especially those who had already been in a disadvantaged position due to a low socioeconomic background or serious health difficulties (e.g., visually impaired students, those with hearing difficulties, or learning difficulties). To tackle these challenges, several adjustments in the educational methods were warranted for cultivating a positive environment at higher education institutions. Hence, the aim of this study was to shed light on the factors that may render university students more susceptible to mental health problems and lower well-being due to the negative consequences of the pandemic as well as to highlight the factors that may empower young people in times of crisis.

Along these lines, the purpose of this study was twofold. First, it aimed at investigating the risk and protective factors of university psychology students’ mental health and well-being during the COVID-19 outbreak, when the first restrictive measures took place in Greece. Second, it aimed at examining how incorporating well-being enhancement into current educational practices may assist higher education students to sustain or improve their mental health and well-being amid the adverse conditions of a crisis, such as the global COVID-19 pandemic. According to the main findings of the study, the pandemic affected different domains of the psychology students’ lives, including their mental health and well-being, their academic studies, and their relationships with others.

In line with the results of previous studies that showed young age to be a main risk factor for being impacted by the pandemic (Ho et al., 2020; Huang and Zhao, 2020; Mazza et al., 2020; Wang et al., 2020b), in our study it was found that increased levels of stress and depression were reported by the youngest university psychology students aged 18–20 years old. Similarly, lower levels of resilience were reported in the age-group of 18–30 years old, while students aged over 40 years old appeared to be more equipped with healthy coping strategies (i.e., cognitive reappraisal) and resilience, and to better sustain their mental well-being during the COVID-19 outbreak. Thus, our study provides further empirical support to World Health Organization’s (2022) clear call for societal systems to develop action plans and age-related interventions for young adults to address mental health issues that have been caused or compounded by COVID-19. Despite previous evidence that showed female students to be more at risk for mental ill health due to the adverse effects of COVID-19 pandemic (e.g., Kaparounaki et al., 2020; Ochnik et al., 2021; Sun et al., 2021; Reyes-Molina et al., 2022), in our study female psychology trainees were found more resilient than male psychology trainees. Yet, it should be noted that female students also reported to use higher levels of expressive suppression to regulate their emotions. Then, more research is needed on the complex cognitive and affective processes that may be important as potential mediators between sociodemographic variables and university students’ mental health.

Based on the findings of our study, the delivery of well-being enhancement interventions within the academic system appears to prevail as a necessity in the contemporary society, as recent research has also underlined (Papadatou-Pastou et al., 2019; Morgan and Simmons, 2021; Villarino et al., 2022). In our study, university psychology students who felt that the quality of their studies had been deteriorated during the lockdown presented increased levels of anxiety. Furthermore, students who were studying at Higher Education for the first time reported increased levels of negative affect, depression, stress, and anxiety, compared to their classmates who were already graduates from other academic fields prior to their current studies. Mental health problems were also found to be associated with the year of studies, since students who were in due graduation reported increased levels of depression and lower levels of resilience than those students who were at earlier stages of their academic career. These findings indicate that should well-being interventions be developed in higher education contexts in times of crisis, special emphasis should be given to those students who are at the “edges of the system,” that is they are either at the first or the last year of their studies. Indeed, being both a beginning student and a graduand represent highly transitional periods in life that pose many personal and social developmental challenges to young people who are struggling to establish their own identity and acquire some sort of environmental mastery. Especially in times of global crisis, the trajectory to adult maturity through the effective adjustment to new environments, such as the transition from high school to the academic community, or from the university to further academic studies or work, may be an overwhelming and stressful experience, given that other hardships (e.g., a socioeconomic crisis as it was the case in Greece) may also occur. The results from the present study highlight the importance to pay special attention to the specific mental health and well-being needs of these particular groups among university students.

Among the prevailing protective factors against the adverse effects of the COVID-19 pandemic were found to be students’ positive relationships with other people. According to the results of our study, high levels of depression were correlated with worse relationships with significant others (i.e., family, friends, and partner), while deterioration of peer relations was associated with increased stress and lower levels of positive affect. On the other hand, gratitude, resilience, and well-being were positively correlated with improved relationships with others, both during and after the lockdown. Additionally, those who were living alone during the quarantine reported higher levels of anxiety, while they employed expressive suppression as an emotion regulation mechanism. In previous studies, positive family relationships were found to ameliorate the negative impact of the pandemic and the subsequent restrictions (Gurvich et al., 2020; López et al., 2020; Zhang and Ma, 2020; Wang et al., 2020a). This study adds to the current body of knowledge by underpinning the importance of sustaining caring and stable relationships not only with family but also with friends to tackle the unprecedented challenges imposed by the pandemic. Furthermore, the unexpected outcome that psychology trainees who lived with their parents after the removal of the restrictive measures reported higher levels of depression, is interesting. Perhaps, COVID-19 related factors, such as the fear of young people of significant others’ health maintenance and their own sense of responsibility to avoid being the agents of COVID-19 infection, as it has been identified elsewhere in the literature (Cao et al., 2020; Chaudhary et al., 2021; Ghazawy et al., 2021; Kibbey et al., 2021; Kim and Kim, 2021; Oh et al., 2021), might explain such an intriguing finding.

The results from this study gear scientific interest in the ways we could develop robust academic communities of mutual and authentic support for young people. Such an endeavor pertains to the argument for a “whole-university” approach (Houghton and Anderson, 2017) where well-being enhancement is embedded into the curriculum along with developing a wider network of support services in the university context. Along these lines, the current study builds on the existing literature by providing preliminary empirical support on the positive impact of such inclusive initiatives that incorporate well-being enhancement as a core ingredient of academic content. According to our findings, psychology students stated decreased levels of depression as well as increased levels of positive affect, satisfaction with life, and emotional and psychological well-being after participating in the current online integrative course that was implemented during the first restrictive measures in Greece. This outcome encourages future research on how effective approaches to cultivating well-being within the university contexts should be developed to incorporate the concept and practices of well-being into the curriculum. Substantial literature exists on the protective role of well-being on mental health (Keyes, 2002, 2005, 2006) and the strong relation of well-being with satisfaction with life (Diener, 2009). The latter was also found in our study as well as a negative association of well-being with depression. Furthermore, psychology trainees’ well-being was found to be determined by students’ experience of gratitude during the restrictions, a feeling that was positively associated with relationships with significant others. Thus, our study adds to the cutting-edge field of implementing online interventions to promote mental health and well-being in Higher Education students (Papadatou-Pastou et al., 2019; Morgan and Simmons, 2021; Villarino et al., 2022) by giving prominence to rather interactive positive components, such as the experience of gratitude, to be included in future well-being enhancement initiatives.

These results suggest that positive psychology factors may have added value to current educational practices for promoting students’ mental health and well-being in times of crisis. Furthermore, online integrative interventions, such as the current one, may be adapted to a non-pandemic situation as a means to tackle the ever-present need for Higher Education students to sustain and improve their mental well-being. Taking into account that mental health of university students has become a priority of vital importance that calls for coordinated action (Auerbach et al., 2018), a whole-setting strategy is recommended to further increase the sustainability of students’ mental health and well-being (Seppälä et al., 2020). Indeed, UK Higher Education institutions have adopted a whole-university strategy with the aim to promote mental health and well-being of students and faculty (UUK, 2020). In line with recent evidence that demonstrated the effectiveness of digital well-being and mental health initiatives in reducing college students’ levels of anxiety and depression (Lattie et al., 2019), and improving their well-being (Winzer et al., 2018; Lattie et al., 2019; Ferrari et al., 2022), further research is deemed necessary to investigate how online holistic interventions, such as the one implemented in our study, may be beneficial for promoting mental health of young people in the post-pandemic era.

There are several notes of caution that should be taken into account in the interpretation of the current findings. Although the research was a pretest-posttest one, this was not based on a randomized two-group control design. Thus, other extraneous factors pertaining to individual or COVID-19 related factors may have potentially affected the outcomes of our research. For example, in the study of Villarino et al. (2022), who evaluated an online well-being program for college students by having an experimental and a control group, no evidence of a significant difference between the experimental participants’ pre-test and post-test PERMA scores were found after the program. However, as the authors underline this was a stand-alone well-being program and more holistic approaches that embed well-being into the curriculum may be more beneficial. Future research including a randomized controlled trial design as well as follow-up measurements is deemed necessary to increase validity of the current preliminary results. Additionally, a larger sample size with a balanced number of female and male students as well as students from different academic fields are all required in future studies to increase the generalizability of research findings. The rather small sample restricted to psychology students in this study does not allow generalization of results to the entire population of university students. In our study, the incorporation of well-being enhancement into the ongoing educational practices was enabled by the fact that the course pertained to clinical psychology issues. Also, the lecturer who ran the course was a trainer of well-being and positive psychology. Thus, it was easier to introduce students to concepts and activities of well-being as an integral part of the course, based on the common theoretical and practical underpinnings in both disciplines (i.e., clinical psychology and positive psychology). Yet, the integration of well-being frameworks into the curricula of different disciplines may be a challenge that will require specific well-being training of the academic staff and wider adjustments of the academic systems to embed a consistent well-being approach across the university processes.

Notwithstanding the aforementioned limitations, the current study contributes to the existing body of knowledge by shedding light on the risk and protective factors of university psychology students’ mental health and well-being amid the COVID-19 pandemic. Also, it provides support for online well-being initiatives to be integrated into the curriculum content and processes at Higher Education as a means to minimize the negative effects caused or exacerbated by the pandemic as well as to maintain and enhance university students’ mental health and well-being in times of adversity.

## Data availability statement

The raw data supporting the conclusions of this article will be made available by the authors, without undue reservation.

## Ethics statement

The studies involving human participants were reviewed and approved by the Ethics Committee of the Panteion University. The patients/participants provided their written informed consent to participate in this study.

## Author contributions

VY conceived, set up and ran the study, and wrote the discussion section and final version of this manuscript. E-KN collected and analyzed the data with the support of EK and GK. E-KN co-wrote the introduction, methods, and results. EK and GK edited the manuscript at different time points. All authors reviewed and edited the final version of the manuscript, read, and approved the final manuscript.

## Conflict of interest

The authors declare that the research was conducted in the absence of any commercial or financial relationships that could be construed as a potential conflict of interest.

## Publisher’s note

All claims expressed in this article are solely those of the authors and do not necessarily represent those of their affiliated organizations, or those of the publisher, the editors and the reviewers. Any product that may be evaluated in this article, or claim that may be made by its manufacturer, is not guaranteed or endorsed by the publisher.

## References

[ref1] Alyoubi A. Halstead E. J. Zambelli Z. Dimitriou D. (2021). The impact of the COVID-19 pandemic on students’ mental health and sleep in Saudi Arabia. Int. J. Environ. Res. Public Health 18:9344. doi: 10.3390/ijerph18179344, PMID: 34501935 PMC8430501

[ref2] Appleby J. A. King N. Saunders K. E. Bast A. Rivera D. Byun J.. (2022). Impact of the COVID-19 pandemic on the experience and mental health of university students studying in Canada and the UK: a cross-sectional study. BMJ Open 12:e050187. doi: 10.1136/bmjopen-2021-050187, PMID: 35074809 PMC8787844

[ref3] Arsandaux J. Montagni I. Macalli M. Texier N. Pouriel M. Germain R.. (2021). Mental health condition of college students compared to non-students during COVID-19 lockdown: the CONFINS study. BMJ Open 11:e053231. doi: 10.1136/bmjopen-2021-053231, PMID: 34413111 PMC8380475

[ref4] Auerbach R. P. Mortier P. Bruffaerts R. Alonso J. Benjet C. Cuijpers P.. (2018). WHO world mental health surveys international college student project: prevalence and distribution of mental disorders. J. Abnorm. Psychol. 127, 623–638. doi: 10.1037/abn0000362, PMID: 30211576 PMC6193834

[ref5] Barros C. Sacau-Fontenla A. (2021). New insights on the mediating role of emotional intelligence and social support on university students’ mental health during COVID-19 pandemic: gender matters. Int. J. Environ. Res. Public Health 18:12935. doi: 10.3390/ijerph182412935, PMID: 34948544 PMC8701843

[ref6] Basson M. J. Rothmann S. (2018). Flourishing: positive emotion regulation strategies of pharmacy students. Int. J. Pharm. Pract. 26, 458–464. doi: 10.1111/ijpp.12420, PMID: 29239045

[ref7] Bjelland I. Dahl A. A. Haug T. T. Neckelmann D. (2002). The validity of the hospital anxiety and depression scale: an updated literature review. J. Psychosom. Res. 52, 69–77. doi: 10.1016/S0022-3999(01)00296-3 11832252

[ref8] Blasco-Belled A. Tejada-Gallardo C. Fatsini-Prats M. Alsinet C. (2022). Mental health among the general population and healthcare workers during the COVID-19 pandemic: a meta-analysis of well-being and psychological distress prevalence. Curr. Psychol. doi: 10.1007/s12144-022-02913-6, [Epub ahead of print] PMC8887799 35250245

[ref9] Boehm J. K. Vie L. L. Kubzansky L. D. (2012). The promise of well-being interventions for improving health risk behaviors. Curr. Cardiovasc. Risk Rep. 6, 511–519. doi: 10.1007/s12170-012-0273-x

[ref10] Bolier L. Haverman M. Westerhof G. J. Riper H. Smit F. Bohlmeijer E. (2013). Positive psychology interventions: a meta-analysis of randomized controlled studies. BMC Public Health 13:119. doi: 10.1186/1471-2458-13-119 23390882 PMC3599475

[ref11] Bonati M. Campi R. Segre G. (2022). Psychological impact of the quarantine during the COVID-19 pandemic on the general European adult population: a systematic review of the evidence. Epidemiol. Psychiatr. Sci. 31:e27. doi: 10.1017/S2045796022000051, PMID: 35475479 PMC9069583

[ref12] Cao W. Fang Z. Hou G. Han M. Xu X. Dong J.. (2020). The psychological impact of the COVID-19 epidemic on college students in China. Psychiatry Res. 287:112934. doi: 10.1016/j.psychres.2020.112934, PMID: 32229390 PMC7102633

[ref13] Carr A. Cullen K. Keeney C. Canning C. Mooney O. Chinseallaigh E.. (2021). Effectiveness of positive psychology interventions: a systematic review and meta-analysis. J. Posit. Psychol. 16, 749–769. doi: 10.1080/17439760.2020.1818807

[ref14] Charles N. E. Strong S. J. Burns L. C. Bullerjahn M. R. Serafine K. M. (2021). Increased mood disorder symptoms, perceived stress, and alcohol use among college students during the COVID-19 pandemic. Psychiatry Res. 296:113706. doi: 10.1016/j.psychres.2021.113706 33482422 PMC7781902

[ref15] Chaudhary A. P. Sonar N. S. Jamuna T. R. Banerjee M. Yadav S. (2021). Impact of the COVID-19 pandemic on the mental health of college students in India: cross-sectional web-based study. JMIRx Med. 2:e28158. doi: 10.2196/28158, PMID: 34606521 PMC8422937

[ref16] Chinna K. Sundarasen S. Khoshaim H. B. Kamaludin K. Nurunnabi M. Baloch G. M.. (2021). Psychological impact of COVID-19 and lock down measures: an online cross-sectional multicounty study on Asian university students. PLoS One 16:e0253059. doi: 10.1371/journal.pone.0253059, PMID: 34343187 PMC8330936

[ref17] Daskalou V. Sigkollitou E. (2012). “Positive and negative affect scale (PANAS)” in Psychometric instruments in Greece. eds. Stalikas A. Triliva S. Roussi P.. 2nd ed (Athens, Greece: Pedio) 750

[ref18] Datu J. A. D. (2018). Flourishing is associated with higher academic achievement and engagement in Filipino undergraduate and high school students. J. Happiness Stud. 19, 27–39. doi: 10.1007/s10902-016-9805-2

[ref19] Diener E. (2009). Assessing well-being: the collected works of Ed Diener (Vol. 39). Netherlands: Springer.

[ref20] Diener E. D. Emmons R. A. Larsen R. J. Griffin S. (1985). The satisfaction with life scale. J. Pers. Assess. 49, 71–75. doi: 10.1207/s15327752jpa4901_13 16367493

[ref21] Elharake J. A. Akbar F. Malik A. A. Gilliam W. Omer S. B. (2022). Mental health impact of COVID-19 among children and college students: a systematic review. Child Psychiatry Hum. Dev. 54, 913–925. doi: 10.1007/s10578-021-01297-1, PMID: 35013847 PMC8747859

[ref22] El-Monshed A. H. El-Adl A. A. Ali A. S. Loutfy A. (2021). University students under lockdown, the psychosocial effects and coping strategies during COVID-19 pandemic: a cross sectional study in Egypt. J. Am. Coll. Heal. 70, 679–690. doi: 10.1080/07448481.2021.1891086, PMID: 33651672

[ref23] Evans S. Alkan E. Bhangoo J. K. Tenenbaum H. Ng-Knight T. (2021). Effects of the COVID-19 lockdown on mental health, wellbeing, sleep, and alcohol use in a UK student sample. Psychiatry Res. 298:113819. doi: 10.1016/j.psychres.2021.113819, PMID: 33640864 PMC9754711

[ref24] Fang Y. Ji B. Liu Y. Zhang J. Liu Q. Ge Y.. (2022). The prevalence of psychological stress in student populations during the COVID-19 epidemic: a systematic review and meta-analysis. Sci. Rep. 12:12118. doi: 10.1038/s41598-022-16328-7, PMID: 35840641 PMC9284967

[ref25] Ferentinos P. Yotsidi V. Porichi E. Douzenis A. Papageorgiou C. Stalikas A. (2019). Well-being in patients with affective disorders compared to nonclinical participants: a multi-model evaluation of the mental health continuum-short form. J. Clin. Psychol. 75, 1585–1612. doi: 10.1002/jclp.22780, PMID: 30995352

[ref26] Ferrari M. Allan S. Arnold C. Eleftheriadis D. Alvarez-Jimenez M. Gumley A.. (2022). Digital interventions for psychological well-being in university students: systematic review and meta-analysis. J. Med. Internet Res. 24:e39686. doi: 10.2196/39686, PMID: 36169988 PMC9557766

[ref27] Fruehwirth J. C. Biswas S. Perreira K. M. (2021). The COVID-19 pandemic and mental health of first-year college students: examining the effect of COVID-19 stressors using longitudinal data. PLoS One 16:e0247999. doi: 10.1371/journal.pone.0247999, PMID: 33667243 PMC7935268

[ref28] Gao J. Zheng P. Jia Y. Chen H. Mao Y. Chen S.. (2020). Mental health problems and social media exposure during COVID-19 outbreak. PLoS One 15:e0231924. doi: 10.1371/journal.pone.0231924, PMID: 32298385 PMC7162477

[ref29] Ghazawy E. R. Ewis A. A. Mahfouz E. M. Khalil D. M. Arafa A. Mohammed Z.. (2021). Psychological impacts of COVID-19 pandemic on the university students in Egypt. Health Promot. Int. 36, 1116–1125. doi: 10.1093/heapro/daaa147, PMID: 33367587 PMC7799058

[ref30] Gokcen N. Hefferon K. Attree E. (2012). University students’ constructions of 'flourishing' in British higher education: an inductive content analysis. Available at: https://internationaljournalofwellbeing.org/index.php/ijow/article/view/84 (Accessed July 20, 2021).

[ref31] Gross J. J. John O. P. (2003). Individual differences in two emotion regulation processes: implications for affect, relationships, and well-being. J. Pers. Soc. Psychol. 85, 348–362. doi: 10.1037/0022-3514.85.2.348, PMID: 12916575

[ref32] Grubic N. Badovinac S. Johri A. M. (2020). Student mental health in the midst of the COVID-19 pandemic: a call for further research and immediate solutions. Int. J. Soc. Psychiatry 66, 517–518. doi: 10.1177/0020764020925108, PMID: 32364039 PMC7405631

[ref33] Gurvich C. Thomas N. Thomas E. H. Hudaib A. R. Sood L. Fabiatos K.. (2020). Coping styles and mental health in response to societal changes during the COVID-19 pandemic. Int. J. Soc. Psychiatry 67, 540–549. doi: 10.1177/0020764020961790, PMID: 33016171

[ref34] Hadjicharalambous D. Demetriou L. Erotokriou K. (2021). Exploring the quality of life and psychological symptoms of university students in Cyprus during the COVID-19 pandemic. J. Soc. Sci. Res. 17, 110–121. doi: 10.24297/jssr.v17i.9112

[ref35] Heffner A. L. Antaramian S. P. (2016). The role of life satisfaction in predicting student engagement and achievement. J. Happiness Stud. 17, 1681–1701. doi: 10.1007/s10902-015-9665-1

[ref36] Hernández-Díaz Y. Genis-Mendoza A. D. Ramos-Méndez M. Á. Juárez-Rojop I. E. Tovilla-Zárate C. A. González-Castro T. B.. (2022). Mental health impact of the COVID-19 pandemic on Mexican population: a systematic review. Int. J. Environ. Res. Public Health 19:6953. doi: 10.3390/ijerph19116953, PMID: 35682536 PMC9180045

[ref37] Ho C. S. Chee C. Y. Ho R. C. (2020). Mental health strategies to combat the psychological impact of COVID-19 beyond paranoia and panic. Ann. Acad. Med. Singap. 49, 1–3. 32200399

[ref38] Hoare E. Bott D. Robinson J. (2017). Learn it, live it, teach it, embed it: implementing a whole school approach to foster positive mental health and wellbeing through positive education. Intnl. J. Wellbeing 7, 56–71. doi: 10.5502/ijw.v7i3.645

[ref39] Holm-Hadulla R. M. Klimov M. Juche T. Möltner A. Herpertz S. C. (2021). Well-being and mental health of students during the COVID-19 pandemic. Psychopathology 54, 291–297. doi: 10.1159/000519366, PMID: 34564075 PMC8678268

[ref40] Houghton A. M. Anderson J. (2017). Embedding mental wellbeing in the curriculum: maximizing success in higher education. J. Higher Educ. 68, 1–43.

[ref41] Howell A. J. (2009). Flourishing: achievement-related correlates of students’ well-being. J. Posit. Psychol. 4, 1–13. doi: 10.1080/17439760802043459

[ref42] Huang Y. Zhao N. (2020). Generalized anxiety disorder, depressive symptoms and sleep quality during COVID-19 outbreak in China: a web-based cross-sectional survey. Psychiatry Res. 288:112954. doi: 10.1016/j.psychres.2020.112954, PMID: 32325383 PMC7152913

[ref43] Huebner E. S. Hills K. J. Jiang X. Long R. F. Kelly R. Lyons M. D. (2014). “Schooling and children’s subjective well-being” in Handbook of child well-being. eds. Ben-Arieh A. Casas F. Frønes I. Korbin J. (Dordrecht: Springer)

[ref44] Kaparounaki C. K. Patsali M. E. Mousa D. P. V. Papadopoulou E. V. Papadopoulou K. K. Fountoulakis K. N. (2020). University students’ mental health amidst the COVID-19 quarantine in Greece. Psychiatry Res. 290:113111. doi: 10.1016/j.psychres.2020.113111, PMID: 32450416 PMC7236729

[ref45] Kavčič T. Avsec A. Kocjan G. Z. (2020). Psychological functioning of Slovene adults during the COVID-19 pandemic: does resilience matter? Psychiatry Q. 92, 207–216. doi: 10.1007/s11126-020-09789-4, PMID: 32556914 PMC7299145

[ref46] Kecojevic A. Basch C. H. Sullivan M. Davi N. K. (2020). The impact of the COVID-19 epidemic on mental health of undergraduate students in New Jersey, cross-sectional study. PLoS One 15:e0239696. doi: 10.1371/journal.pone.0239696, PMID: 32997683 PMC7526896

[ref47] Keyes C. L. (2002). The mental health continuum: from languishing to flourishing in life. J. Health Soc. Behav. 43, 207–222. doi: 10.2307/3090197, PMID: 12096700

[ref48] Keyes C. L. (2005). Mental illness and/or mental health? Investigating axioms of the complete state model of health. J. Consult. Clin. Psychol. 73, 539–548. doi: 10.1037/0022-006X.73.3.539 15982151

[ref49] Keyes C. L. (2006). Mental health in adolescence: is America’s youth flourishing? Am. J. Orthopsychiatry 76, 395–402. doi: 10.1037/0002-9432.76.3.395 16981819

[ref50] Keyes C. L. Wissing M. Potgieter J. P. Temane M. Kruger A. Van Rooy S. (2008). Evaluation of the mental health continuum–short form (MHC–SF) in setswana-speaking south Africans. Clin. Psychol. Psychother. 15, 181–192. doi: 10.1002/cpp.572, PMID: 19115439

[ref51] Kibbey M. M. Fedorenko E. J. Farris S. G. (2021). Anxiety, depression, and health anxiety in undergraduate students living in initial US outbreak “hotspot” during COVID-19 pandemic. Cogn. Behav. Ther. 50, 409–421. doi: 10.1080/16506073.2020.1853805, PMID: 33433271

[ref52] Kim H. R. Kim E. J. (2021). Factors associated with mental health among international students during the COVID-19 pandemic in South Korea. Int. J. Environ. Res. Public Health 18:11381. doi: 10.3390/ijerph182111381, PMID: 34769902 PMC8582877

[ref53] Kim E. S. Kubzansky L. D. Smith J. (2015). Life satisfaction and use of preventive health care services. Health Psychol. 34, 779–782. doi: 10.1037/hea0000174 25420064 PMC4442077

[ref54] Kim H. Rackoff G. N. Fitzsimmons-Craft E. E. Shin K. E. Zainal N. H. Schwob J. T.. (2021). College mental health before and during the COVID-19 pandemic: results from a nationwide survey. Cognit. Ther. Res. 46, 1–10. doi: 10.1007/s10608-021-10241-5, PMID: 34177004 PMC8214371

[ref55] Koelen J. A. Mansueto A. C. Finnemann A. de Koning L. van der Heijde C. M. Vonk P.. (2022). COVID-19 and mental health among at-risk university students: a prospective study into risk and protective factors. Int. J. Methods Psychiatr. Res. 31:e1901. doi: 10.1002/mpr.1901, PMID: 34932250 PMC8886289

[ref56] Kokkinos C. M. Tsouloupas C. N. Voulgaridou I. (2022). The effects of perceived psychological, educational, and financial impact of COVID-19 pandemic on Greek university students’ satisfaction with life through mental health. J. Affect. Disord. 300, 289–295. doi: 10.1016/j.jad.2021.12.114, PMID: 34979179 PMC8733280

[ref57] Koydemir S. Sökmez A. B. Schütz A. (2021). A meta-analysis of the effectiveness of randomized controlled positive psychological interventions on subjective and psychological well-being. Appl. Res. Qual. Life 16, 1145–1185. doi: 10.1007/s11482-019-09788-z

[ref58] Kyriazos T. A. Stalikas A. Prassa K. Galanakis M. Yotsidi V. Lakioti A. (2018a). Psychometric evidence of the brief resilience scale (BRS) and modeling distinctiveness of resilience from depression and stress. Psychology 9, 1828–1857. doi: 10.4236/psych.2018.97107

[ref59] Kyriazos T. A. Stalikas A. Prassa K. Yotsidi V. (2018b). Can the depression anxiety stress scales short be shorter? Factor structure and measurement invariance of DASS-21 and DASS-9 in a Greek, non-clinical sample. Psychology 9, 1095–1127. doi: 10.4236/psych.2018.95069

[ref60] Lamers S. M. Westerhof G. J. Bohlmeijer E. T. ten Klooster P. M. Keyes C. L. (2011). Evaluating the psychometric properties of the mental health continuum-short form (MHC-SF). J. Clin. Psychol. 67, 99–110. doi: 10.1002/jclp.20741, PMID: 20973032

[ref61] Lattie E. G. Adkins E. C. Winquist N. Stiles-Shields C. Wafford Q. E. Graham A. K. (2019). Digital mental health interventions for depression, anxiety, and enhancement of psychological well-being among college students: systematic review. J. Med. Internet Res. 21:e12869. doi: 10.2196/12869, PMID: 31333198 PMC6681642

[ref62] Lee J. Solomon M. Stead T. Kwon B. Ganti L. (2021). Impact of COVID-19 on the mental health of US college students. BMC Psychol. 9:95. doi: 10.1186/s40359-021-00598-3, PMID: 34103081 PMC8185692

[ref63] Leung C. M. Ho M. K. Bharwani A. A. Cogo-Moreira H. Wang Y. Chow M. S.. (2022). Mental disorders following COVID-19 and other epidemics: a systematic review and meta-analysis. Transl. Psychiatry 12:205. doi: 10.1038/s41398-022-01946-6, PMID: 35581186 PMC9110635

[ref64] Li H. Y. Cao H. Leung D. Y. Mak Y. W. (2020). The psychological impacts of a COVID-19 outbreak on college students in China: a longitudinal study. Int. J. Environ. Res. Public Health 17:3933. doi: 10.3390/ijerph17113933, PMID: 32498267 PMC7312488

[ref65] López J. Perez-Rojo G. Noriega C. Carretero I. Velasco C. Martinez-Huertas J. A.. (2020). Psychological well-being among older adults during the COVID-19 outbreak: a comparative study of the young–old and the old–old adults. Int. Psychogeriatr. 32, 1365–1370. doi: 10.1017/S1041610220000964, PMID: 32438934 PMC7324658

[ref66] Lyrakos G. N. Xatziagelaki E. Papazafiropoulou A. K. Batistaki C. Damigos D. Mathianakis G.. (2013). Translation and validation study of the satisfaction with life scale (SWLS) in Greek general population, diabetes mellitus and patients with emotional disorders. Eur. Psychiatry 28:1. doi: 10.1016/S0924-9338(13)76471-X21920709

[ref67] Mazza C. Ricci E. Biondi S. Colasanti M. Ferracuti S. Napoli C.. (2020). A nationwide survey of psychological distress among Italian people during the COVID-19 pandemic: immediate psychological responses and associated factors. Int. J. Environ. Res. Public Health 17:3165. doi: 10.3390/ijerph17093165, PMID: 32370116 PMC7246819

[ref68] McCullough M. E. Emmons R. A. Tsang J. A. (2002). The grateful disposition: a conceptual and empirical topography. J. Pers. Soc. Psychol. 82, 112–127. doi: 10.1037/0022-3514.82.1.112, PMID: 11811629

[ref69] Michopoulos I. Douzenis A. Kalkavoura C. Christodoulou C. Michalopoulou P. Kalemi G.. (2008). Hospital anxiety and depression scale (HADS): validation in a Greek general hospital sample. Ann. General Psychiatry 7:4. doi: 10.1186/1744-859X-7-4, PMID: 18325093 PMC2276214

[ref70] Morgan B. Simmons L. (2021). A ‘PERMA’ response to the pandemic: an online positive education programme to promote wellbeing in university students front. Educ. 6:642632. doi: 10.3389/feduc.2021.642632

[ref71] Mosleh S. M. Shudifat R. M. Dalky H. F. Almalik M. M. Alnajar M. K. (2022). Mental health, learning behaviour and perceived fatigue among university students during the COVID-19 outbreak: a cross-sectional multicentric study in the UAE. BMC Psychol. 10:47. doi: 10.1186/s40359-022-00758-z, PMID: 35236395 PMC8890023

[ref72] Ochnik D. Rogowska A. M. Kuśnierz C. Jakubiak M. Schütz A. Held M. J.. (2021). Mental health prevalence and predictors among university students in nine countries during the COVID-19 pandemic: a cross-national study. Sci. Rep. 11:18644. doi: 10.1038/s41598-021-97697-3, PMID: 34545120 PMC8452732

[ref73] Odriozola-González P. Planchuelo-Gómez Á. Irurtia M. J. de Luis-García R. (2020). Psychological effects of the COVID-19 outbreak and lockdown among students and workers of a Spanish university. Psychiatry Res. 290:113108. doi: 10.1016/j.psychres.2020.113108, PMID: 32450409 PMC7236679

[ref74] Oh H. Marinovich C. Rajkumar R. Besecker M. Zhou S. Jacob L.. (2021). COVID-19 dimensions are related to depression and anxiety among US college students: findings from the healthy minds survey 2020. J. Affect. Disord. 292, 270–275. doi: 10.1016/j.jad.2021.05.121, PMID: 34134025 PMC8595066

[ref75] Otanga H. Tanhan A. Musılı P. M. Arslan G. Buluş M. (2021). Exploring college students’ biopsychosocial spiritual wellbeing and problems during COVID-19 through a contextual and comprehensive framework. Int. J. Ment. Health Addiction 20, 619–638. doi: 10.1007/s11469-021-00687-9, PMID: 34744531 PMC8560020

[ref76] Ouweneel E. Le Blanc P. M. Schaufeli W. B. (2011). Flourishing students: a longitudinal study on positive emotions, personal resources, and study engagement. Int. J. Ment. Health Addict. 6, 142–153. doi: 10.1080/17439760.2011.558847

[ref77] Özdin S. Bayrak Özdin Ş. (2020). Levels and predictors of anxiety, depression and health anxiety during COVID-19 pandemic in Turkish society: the importance of gender. Int. J. Soc. Psychiatry 66, 504–511. doi: 10.1177/0020764020927051, PMID: 32380879 PMC7405629

[ref78] Padrón I. Fraga I. Vieitez L. Montes C. Romero E. (2021). A study on the psychological wound of COVID-19 in university students. Front. Psychol. 12:589927. doi: 10.3389/fpsyg.2021.589927, PMID: 33574786 PMC7870473

[ref79] Papadatou-Pastou M. Campbell-Thompson L. Barley E. Haddad M. Lafarge C. McKeown E.. (2019). Exploring the feasibility and acceptability of the contents, design, and functionalities of an online intervention promoting mental health, wellbeing, and study skills in higher education students. Int. J. Ment. Health Syst. 13:51. doi: 10.1186/s13033-019-0308-5, PMID: 31367229 PMC6647293

[ref80] Pappa S. Chen J. Barnett J. Chang A. Dong R. K. Xu W.. (2022). A systematic review and meta-analysis of the mental health symptoms during the Covid-19 pandemic in Southeast Asia. Psychiatry Clin. Neurosci. 76, 41–50. doi: 10.1111/pcn.13306, PMID: 34704305 PMC8661667

[ref81] Patsali M. E. Mousa D. P. V. Papadopoulou E. V. Papadopoulou K. K. Kaparounaki C. K. Diakogiannis I.. (2020). University students’ changes in mental health status and determinants of behavior during the COVID-19 lockdown in Greece. Psychiatry Res. 292:113298. doi: 10.1016/j.psychres.2020.113298, PMID: 32717710 PMC7357537

[ref82] Pavot W. Diener E. (1993). The affective and cognitive context of self-reported measures of subjective well-being. Soc. Indic. Res. 28, 1–20. doi: 10.1007/BF01086714

[ref83] Pedrelli P. Nyer M. Yeung A. Zulauf C. Wilens T. (2015). College students: mental health problems and treatment considerations. Acad. Psychiatry 39, 503–511. doi: 10.1007/s40596-014-0205-9, PMID: 25142250 PMC4527955

[ref84] Piya F. L. Amin S. Das A. Kabir M. A. (2022). Impacts of COVID-19 on the education, life and mental health of students in Bangladesh. Int. J. Environ. Res. Public Health 19:785. doi: 10.3390/ijerph19020785, PMID: 35055604 PMC8775812

[ref85] Prowse R. Sherratt F. Abizaid A. Gabrys R. L. Hellemans K. G. Patterson Z. R.. (2021). Coping with the COVID-19 pandemic: examining gender differences in stress and mental health among university students. Front. Psych. 12:650759. doi: 10.3389/fpsyt.2021.650759, PMID: 33897499 PMC8058407

[ref86] Qin L.-L. Peng J. Shu M.-L. Liao X.-Y. Gong H.-J. Luo B.-A.. (2023). The fully mediating role of psychological resilience between self-efficacy and mental health: evidence from the study of college students during the COVID-19 pandemic. Healthcare 11:420. doi: 10.3390/healthcare11030420, PMID: 36766995 PMC9914060

[ref87] Reyes-Molina D. Alonso-Cabrera J. Nazar G. Parra-Rizo M. A. Zapata-Lamana R. Sanhueza-Campos C.. (2022). Association between the physical activity behavioral profile and sedentary time with subjective well-being and mental health in Chilean university students during the COVID-19 pandemic. Int. J. Environ. Res. Public Health 19:2107. doi: 10.3390/ijerph19042107, PMID: 35206294 PMC8872099

[ref88] Rogowska A. M. Ochnik D. Kuśnierz C. Chilicka K. Jakubiak M. Paradowska M.. (2021). Changes in mental health during three waves of the COVID-19 pandemic: a repeated cross-sectional study among polish university students. BMC Psychiatry 21:627. doi: 10.1186/s12888-021-03615-2, PMID: 34911485 PMC8672339

[ref89] Sauer N. Sałek A. Szlasa W. Ciecieląg T. Obara J. Gaweł S.. (2022). The impact of COVID-19 on the mental well-being of college students. Int. J. Environ. Res. Public Health 19:5089. doi: 10.3390/ijerph19095089, PMID: 35564484 PMC9100955

[ref90] Seligman M. E. P. (2011). Flourish – a new understanding of happiness and wellbeing – and how to achieve them. London: Nicholas Brealey Publishing.

[ref91] Seppälä E. M. Bradley C. Moeller J. Harouni L. Nandamudi D. Brackett M. A. (2020). Promoting mental health and psychological thriving in university students: a randomized controlled trial of three well-being interventions. Front. Psych. 11:590. doi: 10.3389/fpsyt.2020.00590, PMID: 32760296 PMC7373803

[ref92] Serafini G. Parmigiani B. Amerio A. Aguglia A. Sher L. Amore M. (2020). The psychological impact of COVID-19 on the mental health in the general population. QJM Int. J. Med. 113, 531–537. doi: 10.1093/qjmed/hcaa201, PMID: 32569360 PMC7337855

[ref93] Silișteanu S. C. Totan M. Antonescu O. R. Duică L. Antonescu E. Silișteanu A. E. (2022). The impact of COVID-19 on behavior and physical and mental health of Romanian college students. Medicina 58:246. doi: 10.3390/medicina58020246, PMID: 35208571 PMC8876025

[ref94] Smith B. W. Dalen J. Wiggins K. Tooley E. Christopher P. Bernard J. (2008). The brief resilience scale: assessing the ability to bounce back. Int. J. Behav. Med. 15, 194–200. doi: 10.1080/10705500802222972, PMID: 18696313

[ref95] Smith L. Jacob L. Yakkundi A. McDermott D. Armstrong N. C. Barnett Y.. (2020). Correlates of symptoms of anxiety and depression and mental wellbeing associated with COVID-19: a cross-sectional study of UK-based respondents. Psychiatry Res. 291:113138. doi: 10.1016/j.psychres.2020.113138, PMID: 32562931 PMC7258801

[ref96] Son C. Hegde S. Smith A. Wang X. Sasangohar F. (2020). Effects of COVID-19 on college students’ mental health in the United States: interview survey study. J. Med. Internet Res. 22:e21279. doi: 10.2196/21279, PMID: 32805704 PMC7473764

[ref97] Sun S. Goldberg S. B. Lin D. Qiao S. Operario D. (2021). Psychiatric symptoms, risk, and protective factors among university students in quarantine during the COVID-19 pandemic in China. Glob. Health 17:15. doi: 10.1186/s12992-021-00663-x, PMID: 33494769 PMC7829620

[ref98] Talapko J. Perić I. Vulić P. Pustijanac E. Jukić M. Bekić S.. (2021). Mental health and physical activity in health-related university students during the COVID-19 pandemic. Healthcare 9:801. doi: 10.3390/healthcare9070801, PMID: 34202384 PMC8304952

[ref99] Thakur V. Jain A. (2020). COVID 2019-suicides: a global psychological pandemic. Brain Behav. Immun. 88, 952–953. doi: 10.1016/j.bbi.2020.04.062, PMID: 32335196 PMC7177120

[ref100] Trzebiński J. Cabański M. Czarnecka J. Z. (2020). Reaction to the COVID-19 pandemic: the influence of meaning in life, life satisfaction, and assumptions on world orderliness and positivity. J. Loss Trauma 25, 544–557. doi: 10.1080/15325024.2020.1765098

[ref101] UUK (2020). Step change: mentally healthy universities. Available at: https://www.universitiesuk.ac.uk/what-we-do/policy-and-research/publications/stepchange-mentally-healthy-universities (Accessed March 25, 2023).

[ref102] Van Zyl L. E. Rothmann S. (2012). Flourishing of students in a tertiary education institution in South Africa. J. Psychol. Afr. 22, 593–599. doi: 10.1080/14330237.2012.10820573

[ref103] Villarino R. T. H. Villarino M. L. F. Temblor M. C. L. Chabit N. T. Arcay C. A. L. Gimena G. B.. (2022). Evaluating an online well-being program for college students during the COVID-19 pandemic. J. Health Sci. 12, 74–82. doi: 10.17532/jhsci.2022.1631

[ref104] Visser M. Law-van Wyk E. (2021). University students’ mental health and emotional wellbeing during the COVID-19 pandemic and ensuing lockdown. S. Afr. J. Psychol. 51, 229–243. doi: 10.1177/00812463211012219 PMC8144883 38603064

[ref105] Volken T. Zysset A. Amendola S. Klein Swormink A. Huber M. von Wyl A.. (2021). Depressive symptoms in Swiss university students during the covid-19 pandemic and its correlates. Int. J. Environ. Res. Public Health 18:1458. doi: 10.3390/ijerph18041458, PMID: 33557193 PMC7913894

[ref106] Wang X. Hegde S. Son C. Keller B. Smith A. Sasangohar F. (2020). Investigating mental health of US college students during the COVID-19 pandemic: cross-sectional survey study. J. Med. Internet Res. 22:e22817. doi: 10.2196/22817 32897868 PMC7505693

[ref107] Wang C. Pan R. Wan X. Tan Y. Xu L. Ho C. S.. (2020a). Immediate psychological responses and associated factors during the initial stage of the 2019 coronavirus disease (COVID-19) epidemic among the general population in China. Int. J. Environ. Res. Public Health 17:1729. doi: 10.3390/ijerph17051729, PMID: 32155789 PMC7084952

[ref108] Wang C. Pan R. Wan X. Tan Y. Xu L. McIntyre R. S.. (2020b). A longitudinal study on the mental health of general population during the COVID-19 epidemic in China. Brain Behav. Immun. 87, 40–48. doi: 10.1016/j.bbi.2020.04.028, PMID: 32298802 PMC7153528

[ref109] Watson D. Clark L. A. Tellegen A. (1988). Development and validation of brief measures of positive and negative affect: the PANAS scales. J. Pers. Soc. Psychol. 54, 1063–1070. doi: 10.1037/0022-3514.54.6.1063, PMID: 3397865

[ref110] Werner A. M. Tibubos A. N. Mülder L. M. Reichel J. L. Schäfer M. Heller S.. (2021). The impact of lockdown stress and loneliness during the COVID-19 pandemic on mental health among university students in Germany. Sci. Rep. 11:22637. doi: 10.1038/s41598-021-02024-5, PMID: 34811422 PMC8609027

[ref111] Winzer R. Lindberg L. Guldbrandsson K. Sidorchuk A. (2018). Effects of mental health interventions for students in higher education are sustainable over time: a systematic review and meta-analysis of randomized controlled trials. PeerJ 6:e4598. doi: 10.7717/peerj.4598, PMID: 29629247 PMC5885977

[ref112] World Health Organization. (2022). Mental health and COVID-19: early evidence of the pandemic’s impact. Available at: https://www.who.int/publications/i/item/WHO-2019-nCoV-Sci_Brief-Mental_health-2022.1.

[ref113] Xiong P. Zhang F. Zhang C. Bai J. Luo C. Cao W.. (2021). Factors influencing mental health among Chinese medical and non-medical students in the early stage of COVID-19 pandemic. Front. Public Health 9:603331. doi: 10.3389/fpubh.2021.603331, PMID: 34095044 PMC8172592

[ref114] Xu T. Wang H. (2023). High prevalence of anxiety, depression, and stress among remote learning students during the COVID-19 pandemic: evidence from a meta-analysis. Front. Psychol. 13:1103925. doi: 10.3389/fpsyg.2022.1103925, PMID: 36704682 PMC9871576

[ref115] Yang C. Chen A. Chen Y. (2021). College students’ stress and health in the COVID-19 pandemic: the role of academic workload, separation from school, and fears of contagion. PLoS One 16:e0246676. doi: 10.1371/journal.pone.0246676, PMID: 33566824 PMC7875391

[ref116] Ye B. Zhao S. Zeng Y. Chen C. Zhang Y. (2022). Perceived parental support and college students’ depressive symptoms during the COVID-19 pandemic: the mediating roles of emotion regulation strategies and resilience. Curr. Psychol. doi: 10.1007/s12144-022-03049-3, [Epub ahead of print] PMC8980203 35400981

[ref117] Yotsidi V. (2020). The integration of positive psychology in the clinical milieu: conceptual, empirical and practical implications in the mental health care. Psychology 25, 94–114. doi: 10.12681/psy_hps.25363

[ref118] Yu M. Tian F. Cui Q. Wu H. (2021). Prevalence and its associated factors of depressive symptoms among Chinese college students during the COVID-19 pandemic. BMC Psychiatry 21:66. doi: 10.1186/s12888-021-03066-9, PMID: 33514336 PMC7845579

[ref119] Yusoff M. S. B. (2013). Psychometric properties of the depression anxiety stress scale in a sample of medical degree applicants. Int. Medical J. 20, 295–300.

[ref120] Zhang Y. Ma Z. F. (2020). Impact of the COVID-19 pandemic on mental health and quality of life among local residents in Liaoning Province, China: a cross-sectional study. Int. J. Environ. Res. Public Health 17:2381. doi: 10.3390/ijerph17072381, PMID: 32244498 PMC7177660

[ref121] Zhu Y. Wang H. Wang A. (2021). An evaluation of mental health and emotion regulation experienced by undergraduate nursing students in China during the COVID-19 pandemic: a cross-sectional study. Int. J. Ment. Health Nurs. 30, 1160–1169. doi: 10.1111/inm.12867, PMID: 33848056 PMC8250684

[ref122] Zigmond A. S. Snaith R. P. (1983). The hospital anxiety and depression scale. Acta Psychiatr. Scand. 67, 361–370. doi: 10.1111/j.1600-0447.1983.tb09716.x 6880820

[ref123] Zinchenko Y. P. Shaigerova L. A. Almazova O. V. Shilko R. S. Vakhantseva O. V. Dolgikh. (2021). The spread of COVID-19 in Russia: immediate impact on mental health of university students. Psychol. Stud. 66, 291–302. doi: 10.1007/s12646-021-00610-1 PMC8278806 34276074

